# Subthalamic nucleus deep brain stimulation induces impulsive action when patients with Parkinson’s disease act under speed pressure

**DOI:** 10.1007/s00221-016-4577-9

**Published:** 2016-02-18

**Authors:** Inês Pote, Mariam Torkamani, Zinovia-Maria Kefalopoulou, Ludvic Zrinzo, Patricia Limousin-Dowsey, Thomas Foltynie, Maarten Speekenbrink, Marjan Jahanshahi

**Affiliations:** Unit of Functional Neurosurgery, UCL Institute of Neurology, National Hospital for Neurology and Neurosurgery, 33 Queen Square, London, WC1N 3BG UK; Cognitive Motor Neuroscience Group, UCL Institute of Neurology, London, WC1N 3BG UK; Department of Forensic and Neurodevelopmental Sciences, Institute of Psychiatry, Psychology and Neuroscience, King’s College London, London, SE5 8AF UK; Experimental Psychology, University College London, London, WC1H 0AP UK

**Keywords:** Deep brain stimulation, Impulsivity, Parkinson’s disease, Response threshold, Speed–accuracy trade-off, Subthalamic nucleus

## Abstract

The subthalamic nucleus (STN) is proposed to modulate response thresholds and speed–accuracy trade-offs. In situations of conflict, the STN is considered to raise response thresholds, allowing time for the accumulation of information to occur before a response is selected. Conversely, speed pressure is thought to reduce the activity of the STN and lower response thresholds, resulting in fast, errorful responses. In Parkinson’s disease (PD), subthalamic nucleus deep brain stimulation (STN-DBS) reduces the activity of the nucleus and improves motor symptoms. We predicted that the combined effects of STN stimulation and speed pressure would lower STN activity and lead to fast, errorful responses, hence resulting in impulsive action. We used the motion discrimination ‘moving-dots’ task to assess speed–accuracy trade-offs, under both speed and accuracy instructions. We assessed 12 patients with PD and bilateral STN-DBS and 12 age-matched healthy controls. Participants completed the task twice, and the patients completed it once with STN-DBS on and once with STN-DBS off, with order counterbalanced. We found that STN stimulation was associated with significantly faster reaction times but more errors under speed instructions. Application of the drift diffusion model showed that stimulation resulted in lower response thresholds when acting under speed pressure. These findings support the involvement of the STN in the modulation of speed–accuracy trade-offs and establish for the first time that speed pressure alone, even in the absence of conflict, can result in STN stimulation inducing impulsive action in PD.

## Introduction

The quicker we make a decision, the more likely we are to make errors, whereas the more accurate we try to be, the longer we take. This speed–accuracy trade-off (SAT) is a property of decision-making that can be controlled at will, depending on what is deemed important—be it responding quickly or accurately (Woodworth 1899; Fitts 1966; Wickelgren 1977; Dickman and Meyer 1988). Mathematical models of decision-making propose that when presented with two options, a decision is made only once there is enough evidence to favour one option over another (Vickers 1970; Brown and Heathcote 2008). Starting from baseline, accumulation of information for each option occurs over time. The option that reaches threshold first is selected and executed. In evidence accumulation models, the distance between the baseline and threshold (boundary separation) indicates the amount of information that needs to be accumulated before a decision is made. SAT is controlled by a change in this distance (Ratcliff 1978; Ratcliff and McKoon 2008; Grasman et al. 2009). If the distance is short, the threshold (i.e. decision) is reached quickly, but due to noisy inputs, the probability of it reaching the threshold of an incorrect option first is relatively high. Hence, lower thresholds are generally related to fast, error-prone responding. In contrast, if the distance between the baseline and threshold is large, the threshold is reached more slowly and decisions are made more accurately (Ratcliff and McKoon 2008; Bogacz et al. 2009). According to the ‘STN Theory’ of SAT, ordinarily, in situations of conflict, the STN receives additional excitatory input from the frontal cortex, which raises the response threshold and sends a global ‘no-go’ signal to the output pathways of the basal ganglia, preventing premature responses and allowing time for more information to accumulate before a decision is made (Frank et al. 2007).

In Parkinson’s disease (PD), STN-DBS greatly improves motor symptoms (Deuschl et al. 2006; Weaver et al. 2009; Follett et al. 2010; Williams et al. 2010). In carefully selected PD patients, STN-DBS does not produce any major changes in cognitive function, other than a decline in verbal fluency (Parsons et al. 2006) and deficits of inhibitory and executive control, documented on a range of cognitive and motor tasks (Jahanshahi 2013; Jahanshahi et al. 2015). Psychiatric problems such as euphoria, hypomania, suicidal ideation, apathy and new cases of impulse control disorders (ICDs) have been documented after STN-DBS (Hälbig et al. 2009a, b; Lim et al. 2009; Volkmann et al. 2010; Moum et al. 2012; Castrioto et al. 2014; Hack et al. 2014). While some of these psychiatric problems (e.g. apathy) may mainly relate to post-operative reductions in dopaminergic medication (Thobois et al. 2010; Volkmann et al. 2010; L’Hommée et al. 2012), it is possible that other problems represent STN-induced impulsivity.

Given that some of the psychiatric side effects of STN-DBS are considered to represent stimulation-induced impulsivity, the aim of the present study was to investigate such stimulation-induced impulsivity in an experimental ‘moving-dots’ task. STN-DBS in PD allows for experimental manipulation of STN activity and thus provides a methodology for testing whether the STN modulates SAT and induces impulsivity under speed pressure. In a recent STN-DBS study (Green et al. 2013), the effect of task difficulty was examined by altering the level of stimulus coherence on the ‘moving-dots’ task, with low coherence conditions considered to reflect high conflict. In the present study, however, we maintained the same level of 50 % stimulus coherence, but asked participants (i.e. PD patients and age-matched controls) to complete the perceptual decision-making task, under both speed and accuracy instructions, and on two occasions. The patients completed the task once with STN-DBS on and once with it off, whereas the controls simply repeated the task twice. We applied the drift diffusion model to the data, so as to derive values for response thresholds, drift rates and non-decision times. Both speed instructions (Bogacz et al. 2009) and STN-DBS (McIntyre et al. 2004) are considered to lower activity in the STN itself, which would have implications for the modulation of SAT on this task. The hypothesis being tested was that even in the absence of conflict, when acting under speed instructions, urgency or time pressure would be sufficient to induce lower response thresholds with STN-DBS on versus off, such that patients would respond faster but make more errors.

## Materials and methods

### Participants

In total, 12 PD patients and 12 healthy controls, matched for age (*p* > 0.05) and education (*p* > 0.05), participated. Patients had a clinical diagnosis of idiopathic PD, according to the UK Brain Bank criteria (Starkstein et al. 1992), and had undergone bilateral STN-DBS surgery (time since surgery: *M* = 31.00, SD = 12.16 months). None of the controls had a history of head injury, addiction or neurological and psychiatric disorders. All participants had a normal or corrected-to-normal vision, and they were all right-handed, except for one healthy control. Despite expected group differences, none of the participants were cognitively impaired, clinically depressed or apathetic (Folstein et al. 1975; Starkstein et al. 1992; Beck et al. 1996) (Table 1). The severity of motor symptoms (Unified Parkinson’s Disease Rating Scale, MDS-UPDRS; Goetz et al. 2007) and stage of illness (Hoehn and Yahr Scale; Hoehn and Yahr 1967) were rated by a neurologist with both STN-DBS on and STN-DBS off. Post-operative MRIs verified that at least one of the four electrodes was in or near the sensorimotor dorsal section of the STN in every patient, which was confirmed by a significant beneficial effect on the motor symptoms of PD in each case. Patients were assessed ‘on’ medication, as this was more convenient for them and as dopaminergic medication does not influence performance on the speed–accuracy version of the moving-dots task in PD (Huang et al. 2015). The demographic and clinical details of the samples are presented in Table 1. The study had ethics committee approval, and all participants provided informed consent.

**Table 1:** Demographic details of the healthy controls (HC) and patients with Parkinson’s disease (PD)

	PD	HC	*p* value
Sample size	12	12	−
Gender (male:female)	10:2 (93 % male)	9:3 (75 % male)	0.633
Age (years)	56.75 (5.36)	60.67 (10.58)	0.265
Handedness (RH:LH)	12:0 (100 % RH)	11:1 (92 % RH)	−
Education (years)	14.50 (3.37)	16.96 (3.63)	0.100
MMSE score	28.75 (1.14)	29.83 (0.50)	0.008
BDI score	12.25 (7.45)	3.73 (2.65)	0.002
SAS score	12.58 (5.00)	10.09 (4.01)	0.204
Disease duration (years)	12.58 (3.55)	−	−
Time since DBS surgery (years)	31.00 (12.26)	−	−
Hoehn–Yahr stage (0–5)			
On DBS	2.08 (0.29)	−	−
Off DBS	2.92 (1.24)	−	−
MDS-UPDRS score III (0–132)			
On DBS	30.50 (8.34)	−	−
Off DBS	69.42 (21.03)	−	−

The numbers in parentheses are standard deviations

*RH* right-handed, *LH* left-handed, *MDS-UPDRS* Movement Disorder Society-Unified Parkinson’s disease Rating Scale, *MMSE* mini-mental state examination, *BDI* Beck Depression Inventory, *SAS* Starkstein Apathy Scale

### Design

A repeated-measures design was used. Both patients and healthy controls performed two blocks of the motion discrimination task twice. Patients with PD completed the task once with STN-DBS on and once with STN-DBS off, with the order counterbalanced across patients. The healthy controls also performed the task twice, referred to as Time 1 and Time 2.

### Behavioural task

**Fig. 1:**
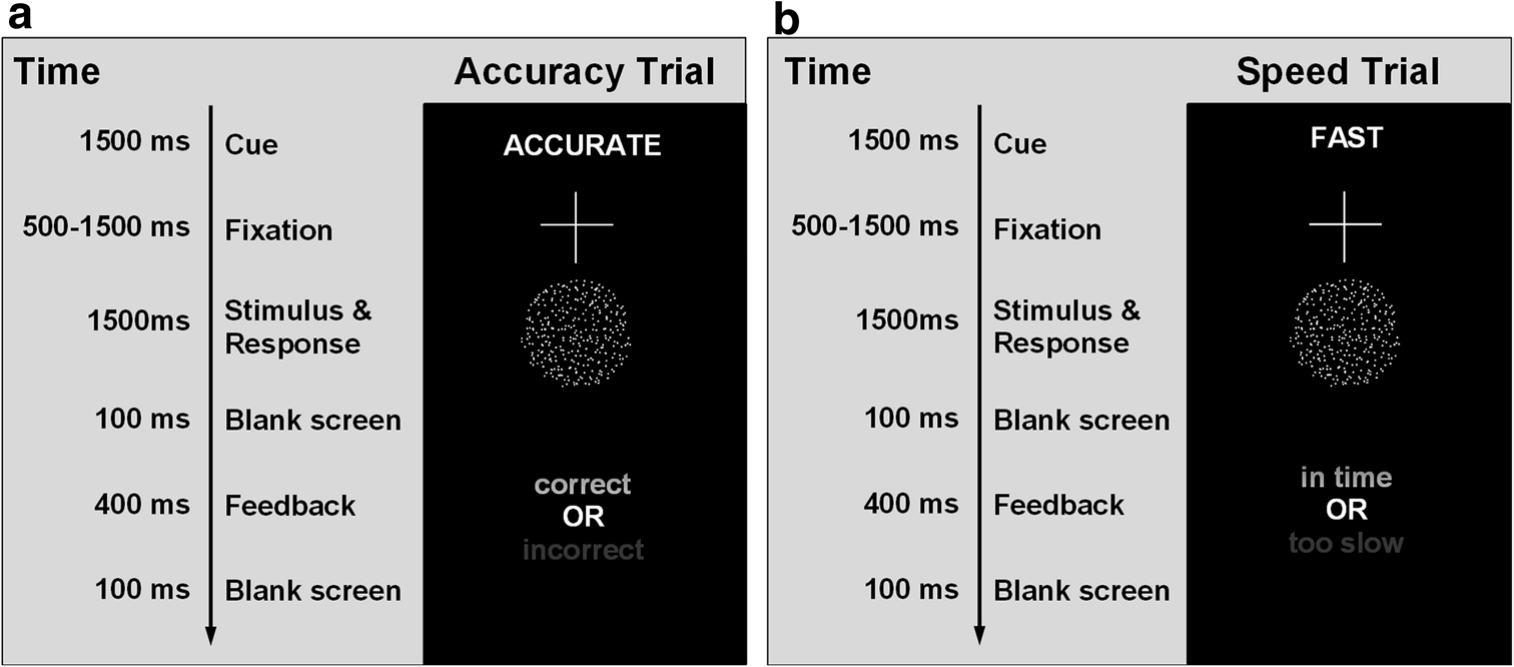
Sequence of stimulus and feedback presentation on the screen for the moving-dots task during: **a** the accuracy trials and **b** the speed trials

The motion discrimination ‘moving-dots’ task (Britten et al. 1992) is a two-choice perceptual decision-making task, which requires participants to decide whether a cloud of dots is moving to the left or the right of the screen (Fig. 1). Out of 120 dots, 50 % moved coherently in one direction and the remaining 50 % moved randomly. Each dot consisted of three pixels, and the diameter of the entire cloud of dots was 250 pixels. Participants indicated their decision by pressing one of two buttons on a custom response box, with either their left (for dots moving left) or right (for dots moving right) index finger. At the start of each trial, a written cue (i.e. FAST for speed and ACCURATE for accuracy), presented for 1500 ms, instructed participants to adopt different levels of cautiousness. The cues were pseudorandomly intermixed, and there were equal numbers of FAST and ACCURATE cues in a block of 200 trials. After each cue presentation, a fixation cross was displayed for a variable time period between 500 and 1500 ms, which introduced temporal unpredictability and ensured that the participants’ attention was focused on the task. Following fixation, the moving-dots were displayed and participants were given a maximum of 1500 ms to view the stimulus and respond. The stimulus disappeared as soon as a response was made and was followed by a blank screen presented for 100 ms.

At the end of each trial, participants received a 400 ms feedback, which depended on the previously presented cue. Under speed conditions, whenever participants exceeded the reaction time criterion of 1500 ms, a ‘too slow’ feedback was presented. If participants responded within the time criterion for the speed condition, they received the feedback ‘in time’. At the end of the accuracy trials, participants were presented with an ‘incorrect’ or ‘correct’ feedback, depending on whether they had made an error or provided a correct response. The negative feedback was presented in red, while the positive feedback appeared in green. Feedback provided an additional incentive for participants to adopt different levels of caution in response to the different cues.

On each occasion, task performance involved two blocks of 200 randomized trials (400 trials in total), with each block containing 100 trials emphasizing speed and another 100 emphasizing accuracy. Completion of each block took about 15 min, and the task was preceded with a practice block of 10 trials to familiarize participants with the task.

### Statistical analysis

Reaction time (RT) and percentage error (PE) were the dependent variables. PE was calculated by taking into consideration the number of non-responses, as follows: PE = [(Total Number of Errors)/(Total Number of Trials − Total Number of Non-Responses)] × 100. Trials with RTs shorter than 200 ms were excluded. There were no significant practice or fatigue effects (*p* > 0.05), and so, the average RTs and PE across the two blocks were calculated (Table 2). Furthermore, the mean RT [*F*(1,11) = 0.018, *p* = 0.895] and PEs [*F*(1,11) = 1.371, *p* = 0.266] of the Time 1 and Time 2 assessments for healthy controls did not differ, indicating that the two assessments were equivalent and could be equated interchangeably with the assessments of STN-DBS on versus off for the patients. The RT and PE data were analysed using two separate repeated-measures analysis of variance (three-way ANOVA), to assess the effects of Group (PD versus controls), STN-DBS/Time (STN-DBS on/Time 1 vs. STN-DBS off/Time 2) and Instruction (Accuracy versus Speed).

**Table 2:** Mean reaction times (RT) and percentage errors (PE) for patients with Parkinson’s disease with subthalamic deep brain stimulation on (STN-DBS on) or off (STN-DBS off), and for healthy controls at the first (Time 1) and second (Time 2) assessments

	STN-DBS on/Time 1		STN-DBS off/Time 2	
	Mean RT (ms)	Mean PE (%)	Mean RT (ms)	Mean PE (%)
Speed instruction				
Parkinson’s disease	485.36 (25.17)	11.86 (1.73)	670.68 (58.15)	7.75 (1.02)
Healthy controls	401.98 (11.46)	6.67 (1.91)	404.86 (9.72)	4.33 (1.11)
Accuracy instruction				
Parkinson’s disease	601.51 (32.22)	3.64 (1.03)	705.06 (54.97)	4.46 (0.84)
Healthy controls	463.08 (21.50)	2.63 (0.69)	461.83 (18.43)	2.54 (0.79)

The numbers in parentheses correspond to the standard error

In addition, a drift diffusion model (Ratcliff 1978; Ratcliff and McKoon 2008) was fitted to both the RTs and errors, to compute boundary separation/response thresholds, drift rates and non-decision times. A free, open-source software was used for the estimation of diffusion model parameters in this study. Using the Fast-dm Software (version 3) (Voss and Voss 2007), the model was estimated separately for each participant and test time, allowing the parameters (response threshold, drift rate, non-decision time and trial-to-trial variability in drift rate and non-decision time) to vary between speed and accuracy instructions. The relative starting point was fixed to zero, which is usual when analysing correct versus incorrect responses. To assess differences between groups and conditions, the parameter estimates were analysed with a linear mixed-effects model, with fixed effects of Time (Time 1 vs. Time 2), Group (PD versus controls), Instruction (Accuracy versus Speed) and STN-DBS (STN-DBS on versus STN-DBS off). The model also included subject-specific random intercepts.

## Results

Compared to when the stimulators were off, STN-DBS resulted in significant improvement (*p* < 0.05) of motor symptoms, with a mean improvement of 56.06 %.

### Group differences in RTs under speed versus accuracy instructions

**Fig. 2:**
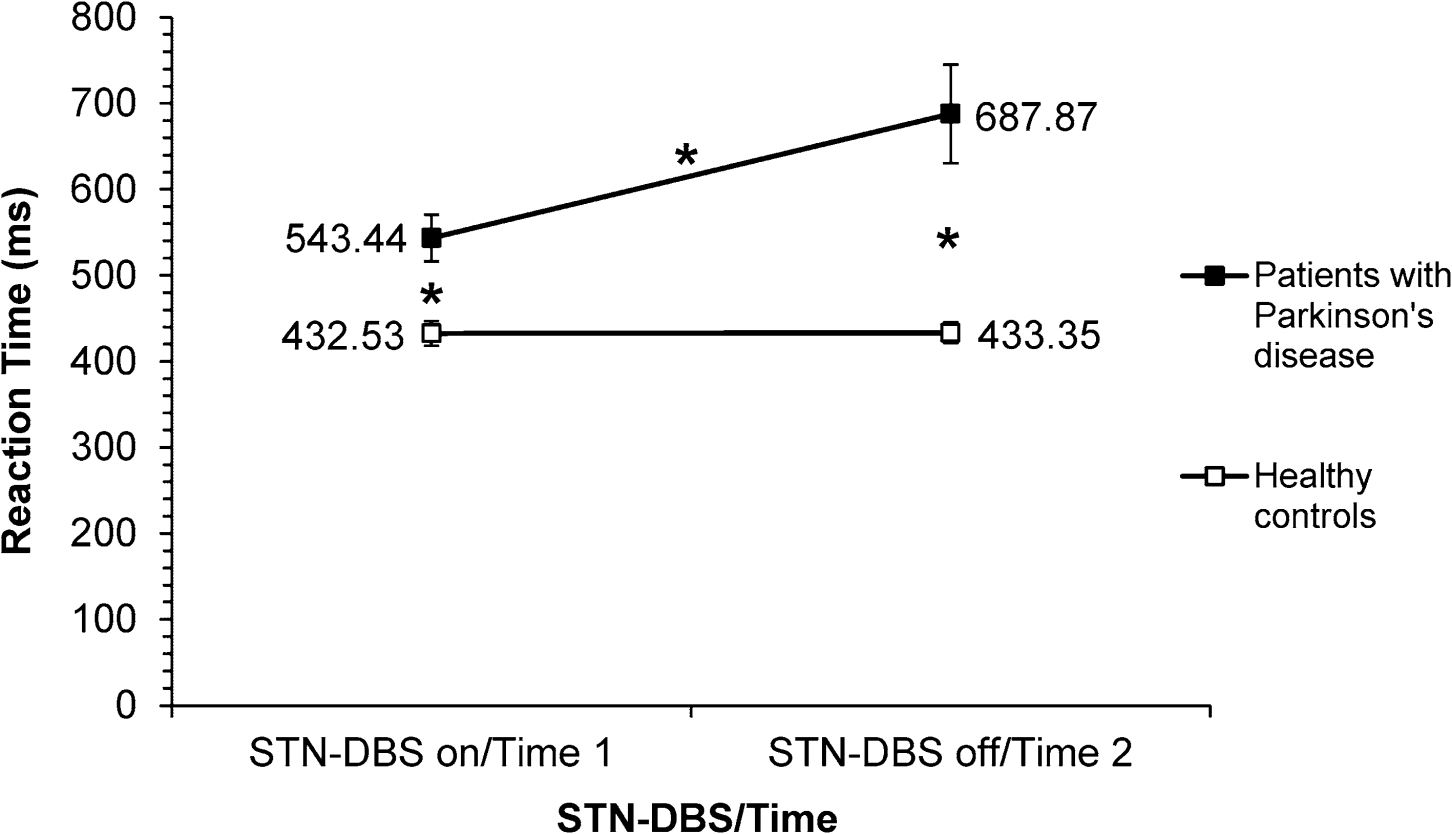
Mean RT (ms) as a function of STN-DBS on or off for patients with Parkinson’s disease, and Time of assessment (Time 1 = first, Time 2 = second assessment) for the healthy controls; *asterisk* denotes significant differences

The RT data for the two groups under speed and accuracy instructions are presented in Table 2. The three-way ANOVA revealed a significant main effect of Group [*F*(1,22) = 19.99, *p* = 0.0001], with the patients responding significantly slower than controls. There was also a significant main effect of STN-DBS/Time [*F*(1,22) = 11.47, *p* = 0.003], indicating that patients with STN-DBS on responded more quickly than with STN-DBS off (Fig. 2).

**Fig. 3:**
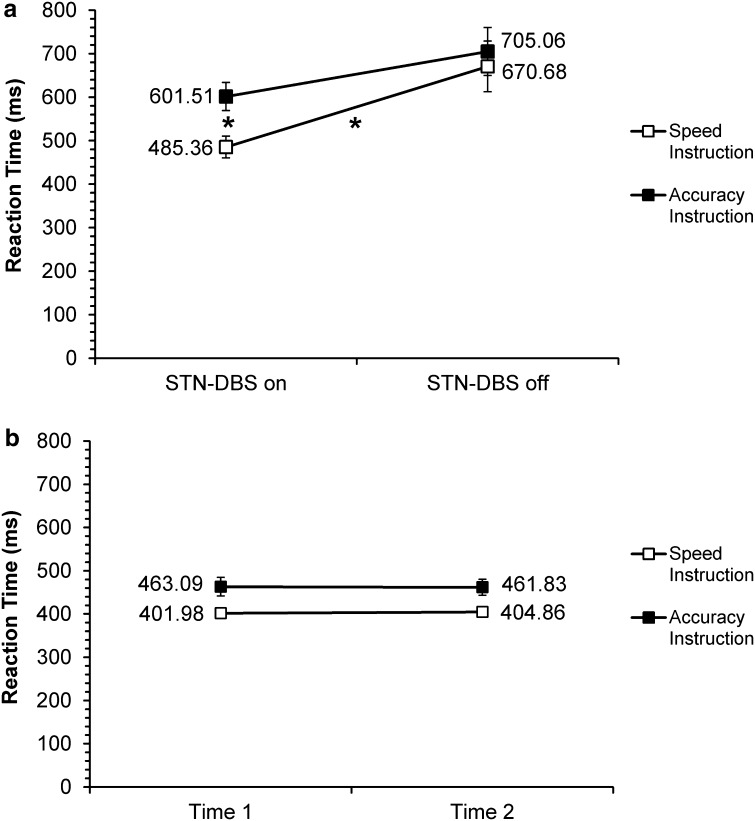
Mean RT (ms) for **a** patients with Parkinson’s disease with STN-DBS on or off and **b** healthy controls for Time 1 (first) and Time 2 (second) assessments, under both speed and accuracy instructions; *asterisk* denotes significant differences

The main effect of Instruction was also significant [*F*(1,22) = 38.08, *p* = 0.0001], suggesting that all participants were conforming to the instruction cues and responding more quickly under speed (490.72 ms), rather than accuracy (557.87 ms) instructions. However, there was also a significant STN-DBS/Time × Instruction interaction [*F*(1,22) = 16.88, *p* = 0.0001], which showed that the effect of instruction was less marked for patients off DBS than on DBS. Most importantly, the Group × STN-DBS/Time × Instruction was found to be significant too [*F*(1,22) = 13.78, *p* = 0.001] (see Fig. 3a, b).

In relation to the significant STN-DBS/Time × Instructions interaction, post hoc tests revealed that while RTs under accuracy instructions between the two assessments were not significantly different [*t*(22) = −1.39, *p* = 0.151], RTs under speed instructions were [*t*(22) = −2.79, *p* = 0.011]. Between STN-DBS on/Time 1 and STN-DBS off/Time 2, a decrease in RT (of 72.63 ms) was noted. This effect was greater for speed (94.10 ms) than accuracy (51.15 ms) instructions. Thus, the source of the STN-DBS/Time × Instruction interaction was the significant difference in RTs under speed instructions. Subsequent post hoc analysis of the significant three-way interaction showed that patients had significantly slower RTs than controls, with both STN-DBS on (Time 1) [*t*(22) = 3.60, *p* = 0.002] and STN-DBS off (Time 2) [*t*(22) = 4.24, *p* = 0.0001]. However, while patients were significantly faster on DBS (543.44 ms) than off it (687.87 ms) (*p* < 0.05), the RTs of the controls did not differ between Time 1 (432.53 ms) and Time 2 (433.35 ms) (*p* > 0.05). Further post hoc analysis revealed that for the patients under accuracy instructions, RTs were not significantly different with STN-DBS on versus STN-DBS off (mean RT difference = 103.55 ms, SD = 78.80) [*t*(22) = −1.63, *p* = 0.118]. In contrast, under speed instructions, RTs were significantly different [*t*(22) = –2.93, *p* = 0.008], with the patients being 185.32 ms (SD = 114.25) faster with stimulation than without (see Fig. 3a).

For the patients, the average magnitude of the difference in RTs for speed versus accuracy instructions was 118.15 ms (SD = 24.43) with DBS on, which was significantly greater [*t*(22) = 4.03, *p* = 0.002] than the speed versus accuracy RT difference of 35.38 ms (SD = 11.01) with DBS off. For the control group, however, the difference in RTs between the two assessments (Time 1 vs. Time 2) was not significant for either speed [*t*(22) = −0.19, *p* = 0.849] or accuracy [*t*(22) = 0.04, *p* = 0.965] instructions (see Fig. 3b). Overall, RTs were significantly altered by STN-DBS on versus off for the patients, but not for Time 1 versus Time 2 for the controls. More importantly, RTs were significantly different for speed and accuracy instructions in the patient group, due to significant speeding of RTs under speed instructions with STN-DBS on.

### Group differences in PEs under speed versus accuracy instructions

**Fig. 4:**
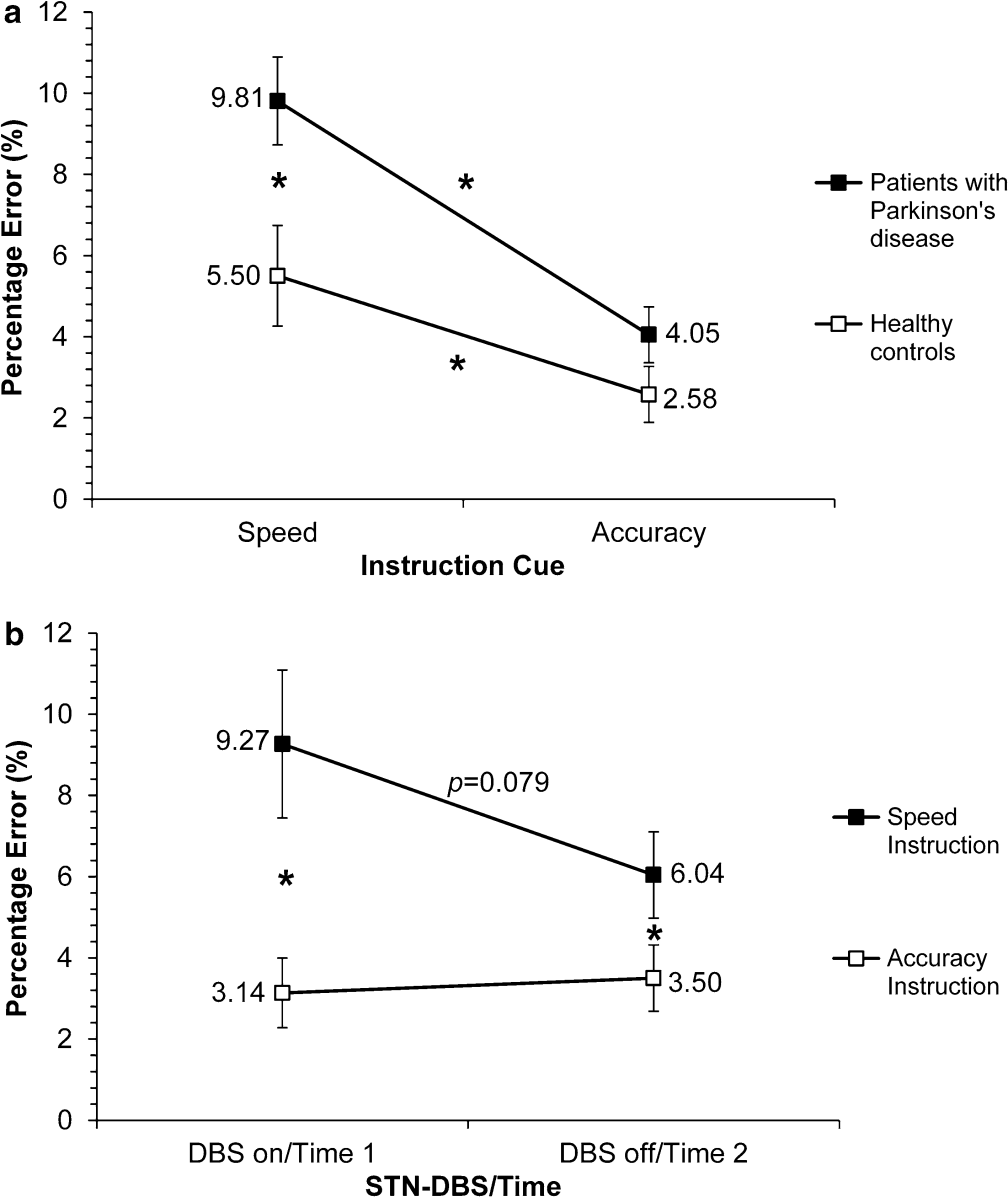
Mean percentage error (PE%) **a** for patients with Parkinson’s disease and healthy controls, under speed versus accuracy instructions, and **b** as a function of STN-DBS/Time, under speed versus accuracy instructions; *asterisk* denotes significant differences

The PE data are presented in Table 2. The three-way ANOVA on PEs revealed a significant main effect of Group [*F*(1,22) = 5.89, *p* = 0.024], with patients making more errors than controls. There was also a significant main effect of Instruction [*F*(1,22) = 44.76, *p* = 0.0001], with participants making more errors under speed than accuracy instructions. The main effect of STN-DBS/Time, however, was not significant [*F*(1,22) = 3.26, *p* = 0.085], suggesting that accuracy rates did not differ between STN-DBS on/Time 1 and STN-DBS off/Time 2. Furthermore, the Group × STN-DBS/Time interaction [*F*(1,22) = 0.08, *p* = 0.785] and the Group × STN-DBS/Time × Instruction interaction were not significant either [*F*(1,22) = 0.95, *p* = 0.341]. However, the Group × Instruction interaction was significant [*F*(1,22) = 4.79, *p* = 0.039] (Fig. 4a), as was the interaction between STN-DBS/Time × Instruction [*F*(1,22) = 6.762, *p* = 0.016] (Fig. 4b).

In relation to the significant Group × Instruction interaction, post hoc tests revealed significant differences in PEs between groups, under speed [*t*(22) = 2.62, *p* = 0.016], but not accuracy [*t*(22) = 1.500, *p* = 0.148] instructions. Furthermore, as indicated by the significant main effect of Instructions, the differences in PE between speed (9.81 %) and accuracy (4.05 %) instructions were significant for the PD patients [*t*(11) = 6.311, *p* < 0.001] as well as the healthy controls (speed: 5.50 % vs. accuracy: 2.58 %) [*t*(11) = 3.166, *p* = 0.009]. For the significant STN-DBS/Time × Instruction interaction, post hoc analysis showed that the PEs under accuracy instructions did not significantly differ between STN-DBS on/Time 1 versus STN-DBS off/Time 2 [*t*(22) = −0.43, *p* = 0.674]. By contrast, under speed instructions, the STN-DBS on/Time 1 (9.27 %) versus STN-DBS off/Time 2 (6.04 %) differences in PEs showed a trend towards significance [*t*(22) = 1.88, *p* = 0.079]. Furthermore, while the speed versus accuracy differences in PEs was significant for both STN-DBS on/Time 1 ([*t*(11) = 5.198, *p* < 0.001] and STN-DBS off/Time 2 [*t*(11) = 4.33, *p* < 0.001], as evident from Fig. 4b, the magnitude of this difference in PE was greater for STN-DBS on/Time 1 (9.27 % with speed vs. 3.14 % for accuracy instructions) than for STN-DBS off/Time 2 (speed 6.04 % vs. 3.50 for accuracy instructions). Thus, the source of the interaction was (1) increased PEs under speed rather than accuracy instructions with STN-DBS on and (2) the differentially greater increase in PEs under speed rather than accuracy instructions with STN-DBS on/Time 1 than with STN-DBS off/Time 2.

In summary, patients made more errors than controls. Patients made more errors under speed instructions than accuracy instructions, and these errors tended to be higher with STN-DBS on, and under speed instructions.

### Drift diffusion model analysis

The boundary separation (*a*) represents the distance between baseline activity and the response threshold to reach a decision. Drift rate (*v*) refers to the speed at which evidence for the correct response accumulates; a high drift rate results in more accurate and faster responses. The non-decision time (*t*_0_) captures the time for stimulus encoding and motor execution.

#### Response threshold

**Fig. 5:**
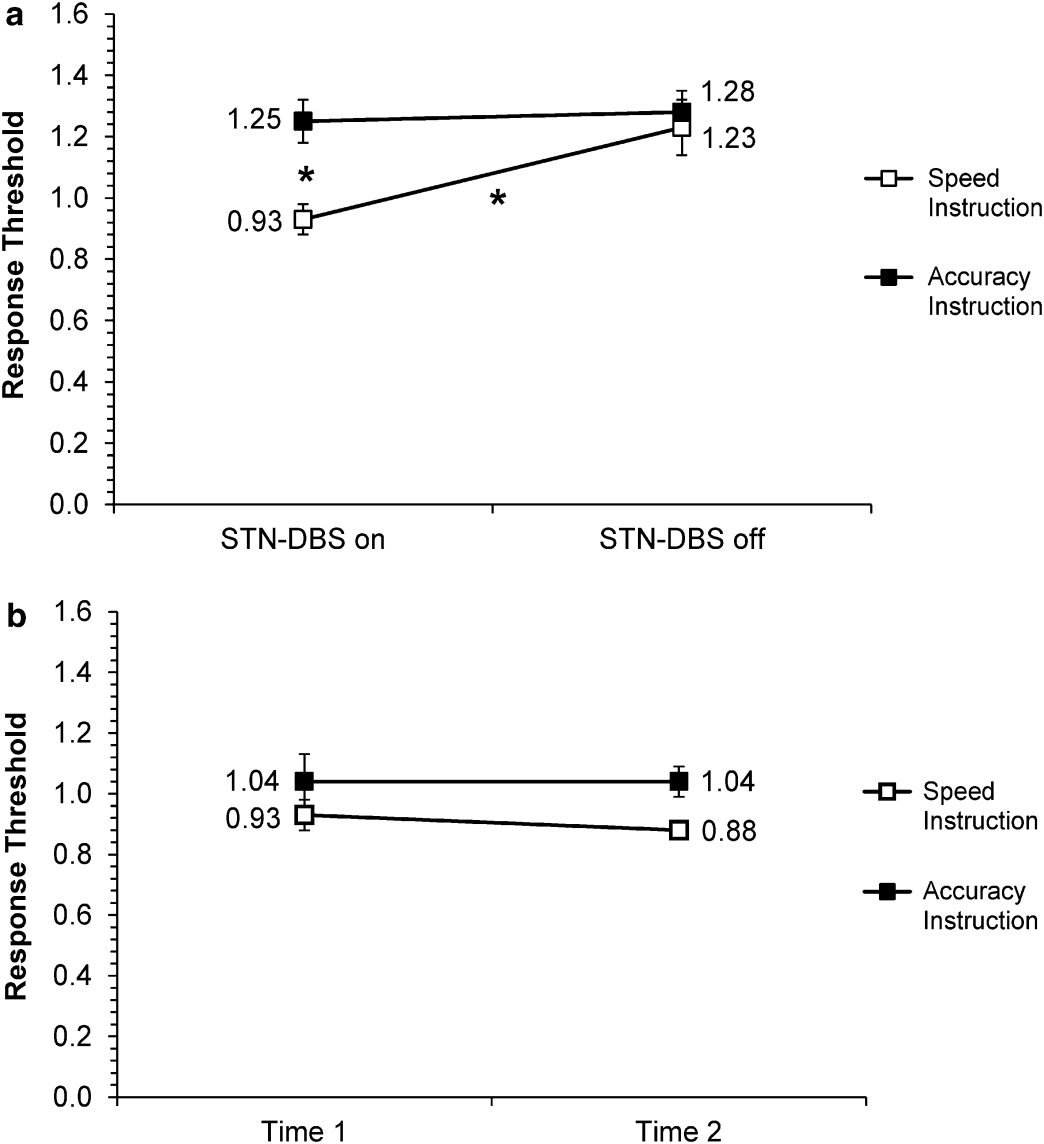
Mean response thresholds for **a** patients with Parkinson’s disease with STN-DBS on or off (DBS on, DBS off), and **b** healthy controls at the two assessments (Time 1 = first, Time 2 = second), under speed versus accuracy instructions

The parameter estimates are presented in Fig. 5a (patients) and Fig. 5b (controls). A significant main effect of Group [*F*(1,21) = 5.71, *p* = 0.026] indicated that patients responded more cautiously than controls. A significant main effect of STN-DBS [*F*(1,63) = 10.62, *p* = 0.002] showed that patients with STN-DBS on had lower response thresholds than with STN-DBS off. The main effect of Instruction was also significant, [*F*(1,63) = 20.64, *p* < 0.001], indicating that participants had lower thresholds and were less cautious under speed instructions. Finally, there was a significant STN-DBS × Instruction interaction [*F*(1,63) = 6.75, *p* = 0.012], indicating that patients with STN-DBS on lowered their thresholds for speed compared to accuracy trials more than with STN-DBS off. Indeed, post hoc tests showed that while patients on DBS had a significantly lower response threshold when acting under speed versus accuracy instructions (*p* < 0.001), this was not the case for patients off DBS (*p* = 0.66). Also, the changes in response thresholds with STN-DBS on versus off were significant for speed instructions (*p* = 0.001), but not for accuracy instructions (*p* = 0.64). No other effects were significance (all *p*s > 0.24).

#### Drift rate

A significant main effect of Group [*F*(1,21) = 18.85, *p* < 0.001] showed that controls had a higher drift rate than patients. The main effect of STN-DBS was marginally significant [*F*(1,63) = 3.85, *p* = 0.054], with patients on DBS showing a higher drift rate. There was a marginally significant main effect of Instruction, [*F*(1,63) = 3.77, *p* = 0.057], indicating a higher drift rate under accuracy, rather than under speed instructions. There was a marginally significant effect of Time [*F*(1,63) = 3.95, *p* = 0.051], with higher drift rates on the second assessment (Time 2). Finally, the Group × Time interaction was significant, [*F*(1,63) = 4.50, *p* = 0.038]; post hoc tests showed that the increase in drift rate with Time was only present for the controls (*p* = 0.007), and not for patients (*p* = 0.99). No other effects approached significance (all *p*s > 0.35).

#### Non-decision time

**Fig. 6:**
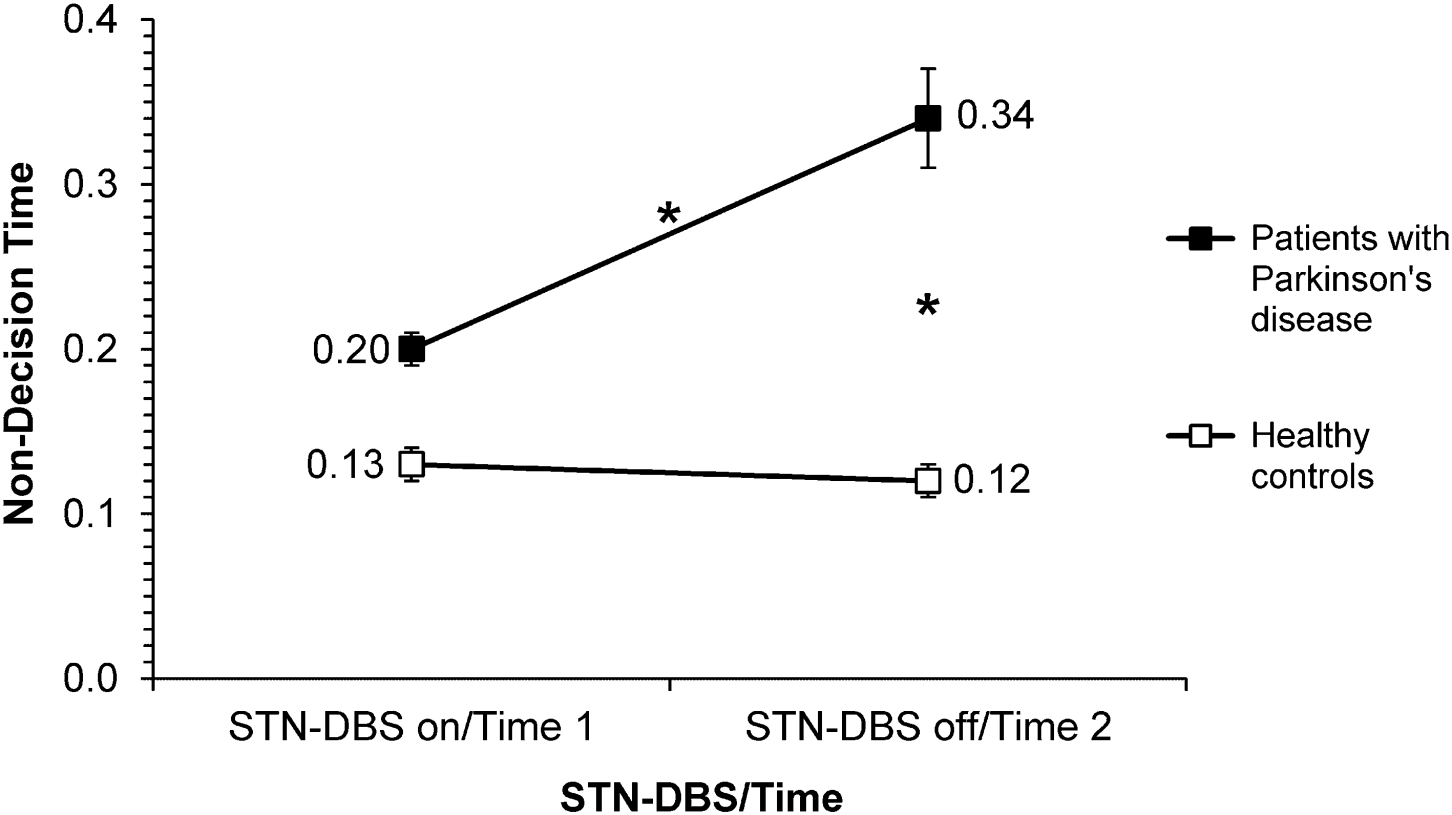
Mean non-decision time for patients with Parkinson’s disease with deep brain stimulation on versus off (DBS on, DBS off) and healthy controls for the Time 1 versus Time 2 assessments (Time 1 = first, Time 2 = second); *asterisk* denotes significant differences

Figure 6 presents the data for non-decision time. A significant effect of Group [*F*(1,21) = 18.85, *p* < 0.001] indicated longer non-decision times for patients than controls. A significant main effect of STN-DBS [*F*(1,63) = 37.52, *p* < 0.001] indicated longer non-decision times for patients with STN-DBS off than on. A significant main effect of Instruction [*F*(1,63) = 16.55, *p* < 0.001] suggested longer non-decision times under accuracy than speed instructions. There was a significant Group × Time interaction, [*F*(1,63) = 6.46, *p* = 0.014]. Post hoc tests showed that while for patients, non-decision times decreased significantly over Time (*p* = 0.012), there was no significant difference between assessment times for controls (*p* = 0.93). Further post hoc tests also showed that while patients on STN-DBS did not differ significantly from the controls on the first assessment (Time 1) (*p* = 0.17), with DBS off, the non-decision times differed significantly from the controls on the second assessment (Time 2) (*p* < 0.001). No other effects approached significance (all *p*s > 0.11).

## Discussion

All participants modulated the speed of their responses according to the instructions. This was reflected in faster RTs, increased PEs and reduced response thresholds, when cued for speed, relative to accuracy. STN-DBS significantly improved the motor symptoms of PD, but it resulted in the performance of the patients to become differentially faster (ΔRT = 185.32 ms) and more erroneous (ΔPE = 4.11 %) when cued for speed, as opposed to accuracy. Furthermore, the response threshold was significantly lower with STN-DBS on versus off, indicating that STN stimulation induced a lowering of the response threshold and a decrease in the level of caution. We conclude that STN stimulation induced impulsive action in patients when they were acting under speed pressure.

### Implications for models of SAT

Imaging studies have clarified the neural correlates of SAT with the pre-supplementary motor area (pre-SMA), anterior cingulate cortex, dorsolateral prefrontal cortex (DLPFC), striatum and the STN, regulating response thresholds and SATs (Heekeren et al. 2006; Forstmann et al. 2008, 2010; Ivanoff et al. 2008; van Veen et al. 2008; Domenech and Dreher 2010; Mansfield et al. 2011; van Maanen et al. 2011; Green et al. 2012; Mulder et al. 2012). Current models of SAT suggest that adjustments of response thresholds are supported by cortico-basal ganglia networks (Bogacz and Turner 2010; Forstmann et al. 2010; Mansfield et al. 2011). The STN receives direct inputs from the pre-SMA, the DLPFC and the anterior cingulate (Afsharpour 1985; Parent and Hazrati 1995; Nambu et al. 1997). It is considered to adjust response thresholds based on the speed or accuracy requirements of a given context, resulting in different levels of response caution. In situations of conflict or when accuracy is imperative, it has been proposed that the STN raises response thresholds and implements a ‘hold your horses’, temporary brake on responding, to ‘buy time’ for accumulation of more information, thus resulting in a more deliberated and cautious, albeit slower response (Frank 2006). Conversely, where speed of responding is emphasized, STN modulation is associated with a lower and a less conservative response threshold and disinhibition of the tonic inhibitory output from the basal ganglia output nuclei to the thalamo-cortical pathways, which facilitates fast but more error-prone responses (Frank 2006). In the light of evidence that the STN is part of an inhibitory network, together with the pre-SMA and the inferior frontal gyrus (Aron et al. 2007; Jahanshahi et al. 2015), it is possible that this threshold modulation function of the STN is interrelated with the STN implementing a temporary brake as part of an inhibitory network.

From experimental manipulation of STN output with the DBS on–off methodology, our study provides more direct and clear evidence in support of the role of the STN in modulating response thresholds and SAT. This was reflected by the finding that under speed instructions, with STN stimulation, patients had differentially and significantly faster RTs and made more errors than with STN-DBS off. These effects were specific to the speed instructions and the PD patients with STN-DBS on, and were not observed with accuracy instructions, for healthy controls or for patients with STN-DBS off. The results of the diffusion model confirmed that, when cued for speed, response thresholds were significantly lower with STN-DBS on than off. Thus, relative to the effect of speed instruction with STN-DBS off, STN stimulation was associated with greater lowering of response thresholds when acting under the urgency of speed pressure. Speed emphasis is predicted to reduce STN activity (Frank 2006; Bogacz et al. 2009), thus resulting in faster and less accurate choices. Stimulation is considered to reduce activity in the STN itself (McIntyre et al. 2004) but to also alter the pattern of pathological oscillatory rhythms (Moran et al. 2012; Whitmer et al. 2012). This reduction in STN activity by STN-DBS coupled with further reduction in STN activity under speed instructions was associated with a significant lowering of response thresholds and fast, errorful choices, as observed by us. Our results support the proposal that the STN and its cortical connections (Frank 2006; Bogacz and Turner 2010) play an important role in setting response thresholds and modulating SAT.

As SAT has been defined as changes in the speed and accuracy of decisions for a *given* task difficulty (Standage et al. 2014), we only investigated the effect of STN-DBS on a ‘moving-dots’ task with a 50 % coherent motion. In a recent STN-DBS study (Green et al. 2013), the effect of task difficulty was examined by altering the level of stimulus coherence on the ‘moving-dots’ task, with low coherence conditions considered to reflect high conflict. STN stimulation reduced the effect of task difficulty on RTs and accuracy, relative to STN-DBS off. Application of a ‘race’ model revealed that STN stimulation altered the patients’ ability to adjust response thresholds for the more difficult low coherence trials. The major significant effect of STN-DBS was on accuracy rather than the speed condition. This is in contrast to our results, with a 50 % coherence condition, where the main effect of STN-DBS was on the speed rather than the accuracy condition, with the patients being faster and less accurate with STN stimulation on versus off when acting under speed pressure. The comparison of the results of the two studies raises interesting questions about the effect of STN-DBS in relation to task difficulty, which may have implications for theories of STN function. As previously noted (Jahanshahi 2013), to date, the STN-DBS-induced deficits in executive and inhibitory control have been mainly observed in conditions of high demand for cognitive control (Hershey et al. 2004; Williams et al. 2015) or motivational salience (Frank et al. 2007). The interaction of STN-DBS with task difficulty is an issue of theoretical and clinical interest that is worth addressing in future studies. Importantly, the current results extend previous findings by demonstrating that when patients were performing an ‘easier’ 50 % coherence version of the moving-dots task, the increased demands of speed pressure induced by the speed instructions was sufficient to result in lowering of response thresholds with STN stimulation, independently of task difficulty or conflict in perceptual decision-making. This is of potential clinical relevance in identifying urgency, speed or time pressure as a factor that may induce impulsive behaviour when patients with STN-DBS make decisions in daily-life situations.

### Implications for STN-DBS in Parkinson’s disease

Our results indicate that with STN-DBS on, PD patients became more impulsive when acting under speed pressure than with STN-DBS off. The important clinical implication of our results is that in real-life situations urgency, time or speed pressure can induce impulsive action in patients who have had STN-DBS.

Impulsivity covers a wide range of inappropriate actions. The main characteristic of impulsive individuals is delay aversion. However, impulsivity is multifaceted and different components of impulsivity have been described (Evenden 1999; Dalley et al. 2011). These include ‘reflection’ impulsivity (acting fast without taking time to reflect), impulsive action (inability to control prepotent responses as reflected by premature responses on go/ no-go RT tasks and failure of motor inhibition on stop signal tasks) and ‘choice’ impulsivity (failure of delayed gratification), which, respectively, operate at the preparation, execution and outcome stages of behavioural control (Cavanagh et al. 2014). These different components of impulsivity are likely to have distinct neurobiological substrates (Dalley et al. 2011; Dalley and Roiser 2012) and STN-DBS is likely to only affect specific components of impulsivity. While there is evidence for STN-DBS-induced impulsive action (Jahanshahi et al. 2000; Hershey et al. 2004; Witt et al. 2004; Frank et al. 2007; Ballanger et al. 2009; Ray et al. 2009; Hershey et al. 2010; Wylie et al. 2010; Cavanagh et al. 2011; Obeso et al. 2013; Plessow et al. 2014), there is no or scant evidence supporting an effect of STN stimulation on other components of impulsivity, relating to reflection impulsivity, delay aversion or risk-taking (Oyama et al. 2011; Torta et al. 2012; Djamshidian et al. 2013). Therefore, not all forms of impulsivity are detrimentally affected by STN-DBS in PD. The effect of STN-DBS on the ability to delay gratification remains to be examined. The present results extend this evidence by demonstrating that STN stimulation is associated with lower response thresholds, conducive to impulsivity and less cautious responding, relative to STN-DBS off when patients make decisions under speed pressure, even in the absence of conflict.

STN-DBS can be associated with psychiatric side effects, such as hypomania, pathological crying and mirthful laughter, representing disinhibition (Castrioto et al. 2014; Volkmann et al. 2010) and post-surgical development of new cases of ICDs (Hälbig et al. 2009a, b; Lim et al. 2009; Moum et al. 2012; Hack et al. 2014). What remains unclear is whether the STN stimulation-induced impulsivity observed by us relates to, or plays a causal role in some of these psychiatric side effects, which have also included attempted and completed suicide in a minority of operated patients (Soulas et al. 2008; Voon et al. 2008). This is a question to be addressed in future studies.

## Abbreviations

DBS: Deep brain stimulation
DLPFC: Dorsolateral prefrontal cortex
ICD: Impulse control disorder
PD: Parkinson’s disease
PE: Percentage errors
pre-SMA: Pre-supplementary motor area
SAT: Speed–accuracy trade-off
STN: Subthalamic nucleus
STN-DBS: Deep brain stimulation of the subthalamic nucleus
RT: Reaction time
